# Factors predicting symptoms of somatization, depression, anxiety, post-traumatic stress disorder, self-rated mental and physical health among recently arrived refugees in Germany

**DOI:** 10.1186/s13031-020-00291-z

**Published:** 2020-07-09

**Authors:** Yuriy Nesterko, David Jäckle, Michael Friedrich, Laura Holzapfel, Heide Glaesmer

**Affiliations:** Department of Medical Psychology and Medical Sociology, University of Leipzig, Philipp-Rosenthal-Str. 55, 04103 Leipzig, Germany

**Keywords:** Refugee, PTSD, Depression, Anxiety, Somatization, Asylum

## Abstract

**Background:**

There is a large body of research indicating increased prevalence rates of mental disorders among refugees. However, the vast majority of the evidence available on risk factors for mental disorders among refugees focuses on post-migration stressors and was collected in surveys that were conducted months and sometimes years after the participants had resettled.

**Objective:**

In the present study, we analyze socio-demographic and flight-related characteristics as predictors for symptoms of somatization, depression, anxiety, and post-traumatic stress disorder as well as self-rated mental and physical health in recently arrived refugees (up to 4 weeks after arrival) in Germany.

**Methods:**

The study was conducted in a reception facility for asylum-seekers in Leipzig, Germany. A total of 1316 adult individuals arrived at the facility during the survey period; 502 took part in the study. The questionnaire (self-administrated) included socio-demographic and flight-related questions as well as standardized instruments for assessing PTSD (PCL-5), depression (PHQ-9), anxiety (HSCL-10) and somatization (SSS-8). Linear regression models were conducted to predict symptoms of different mental disorders as well as self-rated mental and physical health.

**Results:**

Lack of information about family members and subjective need for health care were found to be significantly associated with symptoms of depression, somatization, anxiety, and PTSD. Better self-rated mental health was significantly associated with partnership, childlessness, lower number of traumatic events, and having information about family left behind. No associations were found between flight-related factors and symptom burden.

**Conclusions:**

The results provide initial methodologically robust insights for research and health care services, which should aid in better identifying newly arrived refugees in need of psychosocial care. Furthermore, the results might help answering the question of how to provide health care for highly vulnerable groups within refugee populations regardless their residential status.

## Background

The number of people who have been forcibly displaced by armed conflicts, political instability and/or economic crises in different parts of the world has been growing for years. In total, millions of people have left their homes and sought asylum in neighboring countries, a relatively small proportion have arrived in high-income Western countries [1]. In light of this, there is no doubt that the wide spectrum of different adverse and/or stressful events most refugees experience before and while leaving their homes is conducive to the development of mental disorders [2], reflecting in many cases high level of human rights violations across the globe. Available evidence on mental health in refugees generally focuses on (I) prevalence rates of different mental disorders, (II) risk factors for developing mental disorders with respect to the process of fleeing, and (III) development and/or evaluation of treatment programs for those in need [3]. In the present study, we focus mainly on possible risk factors for mental distress in newly arrived refugees in Germany considering symptom burden as well as prevalence rates for different mental disorders, reported in detail elsewhere [4].

In general, there is a large body of evidence indicating significantly increased prevalence rates of mental disorders among refugees compared to both native and other migrant populations. Of these disorders, post-traumatic stress disorder (PTSD) and depression have been the most frequently investigated [5–7]. However, a wide range of prevalence rates for PTSD and depression in different refugee populations has been reported during the last decade (e.g. 0–99% for PTSD and 3–85% for depression [8]). Studies reporting robust epidemiological data on mental health among recent refugee populations are still rare [3]. For example, in a 2017 population-based survey by Tinghög et al. [9], weighted prevalence rates of 40.2% for depression, 31.8% for anxiety, and 29.9% for PTSD were reported in Syrian refugees (*N* = 1215) who had resettled in Sweden; Steel et al. [10] reported prevalence rates of 47% for PTSD and 20% for depression in refugees from predominantly Sub-Saharan Africa (*N* = 420), using stratified quota sampling based on Swedish census data. All in all, it can be assumed that about half of all refugees arriving in Western high-income countries suffer from at least one mental disorder [3, 4].

**Fig. 1:**
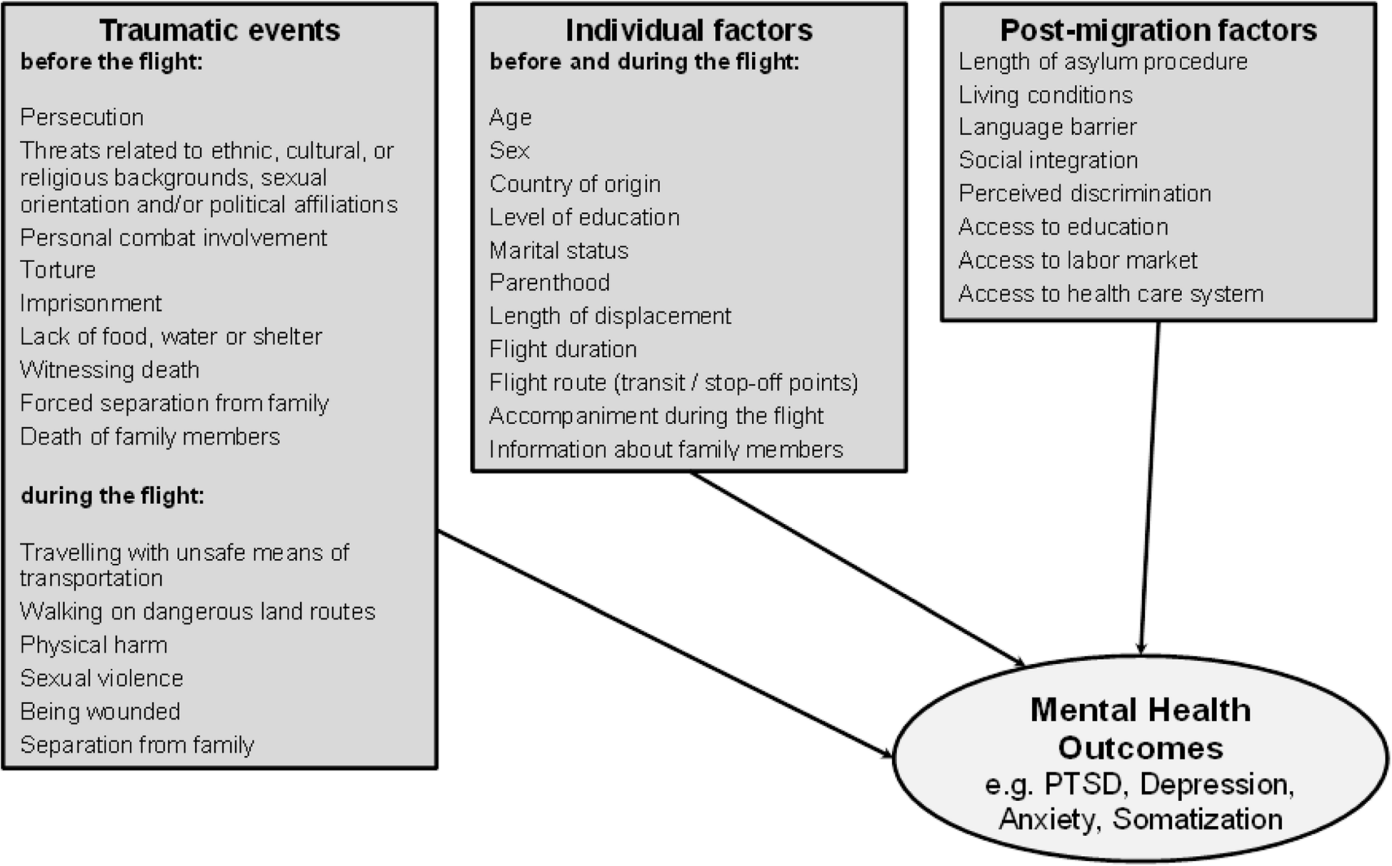
Risk factors for mental disorders in refugee populations

In contrast to research that have focused on prevalence rates, there are further studies that provide more evidence on specific risk factors for mental disorders. These are characterized however by a variety of methodological specifications, which ultimately hamper their comparability (e.g. sample size, sampling methods, selection bias, instruments used, time of assessment etc.). Nevertheless, it is still possible to break often investigated risk factors for mental disorders among refugees into three main categories: (I) traumatic events experienced before or during the flight, (II) individual factors before and/or during the flight (e.g. socio-demographic characteristics, flight duration and/or length of displacement, accompaniment during the flight), and (III) post-migration experiences, as illustrated in Fig. 1 [3, 11–13].

As mentioned above, multiple potential traumas experienced before flight such as persecution, threats related to an individual’s ethnic, cultural, or religious background, sexual orientation, and/or political affiliations, personal combat involvement, torture, imprisonment, lack of food, water or shelter, witnessing death, forced separation from family, and death of family members are the most common reasons people flee their home country. Moreover, numerous severe traumatic experiences refugees are likely to face while fleeing have been reported by previous research, e.g.: travelling in unsafe means of transportation, walking on dangerous land routes, experiences of physical harm, sexual violence and exploitation, being wounded, being separated from family members, or witnessing the loss of loved ones. In many cases, these experiences are linked to human trafficking [6–8, 14].

In general, the higher the number of traumatic events a person experiences, the more vulnerable he or she is to developing mental disorders. Compared to non-interpersonal traumatic events, interpersonal traumatic events in particular are more likely to lead to a higher symptom burden [15] of more severe forms of PTSD [16] as well as depression [17]. Moreover, increased suicidality is reported in those affected by interpersonal trauma [18]. The impact of interpersonal traumatic events on mental health symptoms in refugees has been shown in numerous studies [19–21].

The individual factors ‘*age at arrival’* and ‘*sex’* have often been analyzed as possible predictors for mental health in different refugee populations. With respect to ‘*age at arrival’*, there are inconsistent results. Some studies report better mental health in younger refugees [22–25], while other studies indicate no age-related differences [26] or clear evidence of age as an influential factor [14]. There are consistent reports indicating higher levels of symptom burden in female refugees that are linked with inherent differences between males and females in general [25, 27–30].

However, the vast majority of the evidence available on risk factors for mental disorders among refugees focuses on post-migration stressors and was collected in surveys that were conducted months and sometimes years after the participants had resettled. According to a recently published review by Giacco, Laxhman and Priebe [3], mental distress in refugees after resettlement is positively associated with the length of their asylum procedure, poor living conditions, social isolation, unemployment, and acculturation difficulties that stand in the way of their successful integration and accessing mental health care in their host country (e.g. lack of knowledge about local health care systems, distrust in public organizations, and different beliefs about and consequently representations of psychological symptoms).

It is important to emphasize the necessity to see the differentiation between pre-, peri-, and post-migratory traumatic experiences as rather conceptual (e.g. [31]): The adverse events we mention might often be cross-categorical and long-term events interacting with each other. Moreover, the list of events in Fig. 1 is not exhaustive; especially due to post-migration stressors being investigated previously [32]. The present study focuses on potential risk factors for mental distress in newly arrived refugees (up to 4 weeks after arrival), considering the time frame of symptom burden (e.g. last 7 days, last 2 weeks and/or last 4 weeks) as linked to pre- and/or peri-migration experiences and thus not including long-term post-migration stressors in our analyses.

To the best of our knowledge, the present study is the first to provide robust epidemiological data (for more detailed information on the study protocol refer to [4]) with a focus on socio-demographic and flight-related characteristics as possible predictors for different mental disorders among recently arrived refugees. At the same time, it is worth noting that only studying refugees who have just arrived could result in the impact of post-migration stressors going un- or underreported. Furthermore, there are no methodically sound studies available that report on self-rated health status among refugees. Especially considering the possible development of psychosocial and/or psychotherapeutic interventions, data on subjective health in different groups of refugees right after arrival are an important and necessary source of information for both clinicians and policy makers.

Therefore, the rationale of the present study was to analyze socio-demographic and flight-related characteristics as possible predictors for symptoms of somatization, depression, anxiety, and post-traumatic stress disorder as well as self-rated mental and physical health in recently arrived refugees in Germany based on survey data assessed using an epidemiological approach.

## Methods

### Data collection and study sample

This study was conducted between May 2017 and June 2018 in a primary reception facility operated by the Federal State of Saxony for asylum-seekers in Leipzig, Germany. The study’s target population consisted of adult individuals (≥18 years) residing in the facility during the survey period, with no additional exclusion criteria defined for the recruitment procedure. However, during the recruitment language skills in the following languages become criteria of inclusion: Albanian, Arabic, English, Farsi, French, German, Kurdish, Russian, Spanish, Tigrinya, Turkish and Urdu.

**Fig. 2:**
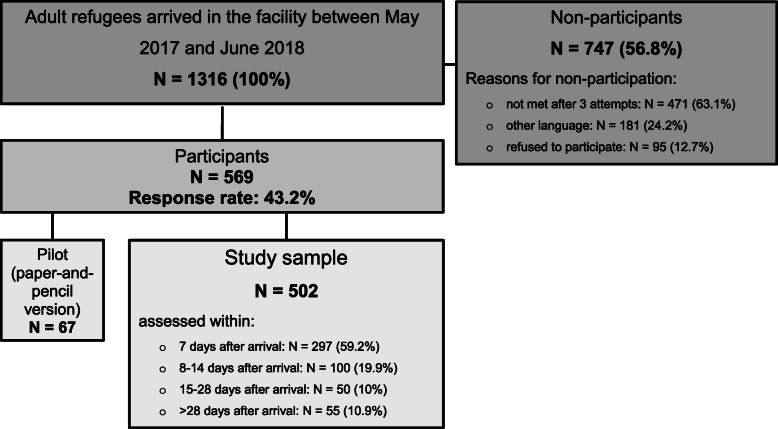
Study procedure

Usually refugees arriving in the facility apply for asylum within the first 2 weeks. Thus, formally all participants of the present study will become asylum-seekers, but due to the date of participation some of them were not asylum-seekers yet. We use the term ‘refugees’ to include all participants. Figure 2 gives an overview of the study procedure.

Based on the facility’s register of all newly arrived residents, potential study participants were approached by members of the project staff in their residential unit, informed about the study objectives as well as data protection policy, and, in the event that they were willing to participate, introduced to the survey procedure. Between May 1st and May 15th of 2017, the participants were asked to fill out a paper version of the questionnaire (pilot study; *N* = 67) to prove the usability of the instruments; after May 17th of 2017, the participants filled out a tablet-based questionnaire in their native language. After information sheets were handed out and consent to participate was given, the participants responded to the questionnaire by themselves (time needed: approximately 45 min). Project staff was available to answer questions when necessary covering some languages spoken by the residents (e.g. Arabic, Farsi, English, French, Russian, Spanish and Turkish). The assessments took place three times a week, on Mondays, Wednesdays, and Thursdays between 10 a.m. and 1 p.m. Data were electronically transferred and administered consecutively to the ongoing data collection using LimeSurvey Offline-App for android systems. Data control and consistency checks were carried out at monthly intervals and a simple plausibility check was carried out immediately after the entry of a maximum of 30 data sets. Data were stored in anonymous form on a computer at the University of Leipzig network in accordance with the data protection guidelines.

A total of 1316 adult refugees were newly accommodated in the primary reception facility during the survey period, 569 of whom took part in the study. Of these, 67 individuals filled out the paper version of the questionnaire (pilot study) and 502 (study sample) responded via tablet (response rate 43.2%). Within these, about 60% (*n* = 297) were assessed during the first 7 days after the arrival, another 19.9% (*n* = 100) during the second week (between 8 and 14 days) after the arrival, 10% (*n* = 50) during the period of 15–28 days after the arrival, and finally 10.9% (*n* = 55) > 28 days after the arrival. The majority of non-participants (63.1%; *n* = 417) were residents who could not be contacted after three attempts to visit them, 24.2% (*n* = 181) could not be included due to their native language, and 95 individuals (12.7%) refused participation. Data on all non-participants’ age, sex, and country of origin were recorded to identify possible selection bias. Detailed information on age, sex and country of origin of non-participants as well as calculated non-response weights were published previously [4].

The study was approved by the Ethics Committee of the Medical Faculty of the University of Leipzig (446/16-ek). All study procedures were conducted in accordance with the Helsinki Declaration and its later amendments or comparable ethical standards. Written informed consent was obtained from all study participants.

### Measures

The questionnaire used in the present study included socio-demographic and flight-related questions, standardized instruments for assessing PTSD, symptoms of depression, anxiety and somatization as well as questions for assessing self-rated mental and physical health. The German version of the questionnaire (both paper and pencil as well as tablet-version) was translated and back-translated into 10 different languages (Albanian, Arabic, English, Farsi, French, Kurdish, Russian, Spanish, Turkish and Urdu) according to the proportion of refugees arrived in Germany during the year preceding the survey by a professional translation agency (mt-g medical translation GmbH) specialized in medical translations. The Tigrinya version of the questionnaire was translated and back-translated by the same agency based on the English version of the questionnaire. All back-translations were reviewed by the first and last author and, when necessary, returned to the agency for final modification/adjustment.

#### Sociodemographic and flight-related characteristics

Participants were asked to provide information about their age, sex, country of origin, marital status, number of children, level of education, last occupation, duration of their flight, accompaniment during the flight, and current access to information about family members who were left behind, as well as present need for support and/or assistance due to the asylum procedure, family reunion request, and/or health care system. Using the global peace index [33], which indicates the relative level of peacefulness in specific nations and regions, a metric variable was built based on participants’ countries of origin in order to reflect possible impacts of the conditions in each country of origin on their levels of mental disorder symptom burden.

#### Traumatic events

Traumatic events were assessed using the DSM-5 Life Events Checklist (LEC-5) for assessing trauma exposure [34]. The LEC-5 is comprised of 16 items, which address different types of events that can potentially result in PTSD or distress. The following response categories are given for each type of event: ‘1’ happened to me, ‘2’ witnessed it, ‘3’ learned about it, ‘4’ part of my job, ‘5’ not sure, and ‘6’ doesn’t apply. The number of events reported by participants as ‘happened to me’ was summed up to the category number of traumatic events. In addition, the category experience of at least one interpersonal traumatic event was calculated by considering the events – physical assault, assault with a weapon, sexual assault, other unwanted sexual experience, captivity and serious injury, harm or death being caused by the participant to someone else – being answered as ‘happened to me’. In the analysis presented here, we decided to consider the category “happened to me” only to focus on the very strict definition of trauma exposure.

#### Post-traumatic stress disorder

PTSD was assessed with the PCL-5 (PTSD-Checklist), a 20-item self-report instrument, which assesses symptoms of PTSD as defined by the DSM-5 [35]. The 20 items of the PCL-5 reflect the frequency with which respondents have experienced the item in question rated on a 5-point Likert-scale ranging from ‘not at all’ to ‘extremely’. A total score (0–80) can be obtained by summing up the scores for each of the 20 items. A score at or above the cut-off score of 33 indicates the presence of PTSD in the respondent. Cronbach’s α in the present study was α = .95 (.93 to .97. for the different language versions).

#### Anxiety

Symptoms of anxiety were assessed with the anxiety-subscale of the Hopkins Symptom Checklist (HSCL-25 [36]). The HSCL-25 anxiety-subscale consists of 10 items assessing symptoms experienced within the last week on a 4-point Likert-scale, anchored ‘not at all’, ‘a little’, ‘quite a bit’, and ‘extremely’. As recommended by several studies that have used the HSCL-25 to assess anxiety in different refugee populations [37], individuals with a mean score of 1.75 or higher were classified as suffering from clinically relevant symptoms of anxiety. The internal consistency of anxiety-subscale scores across the study was α = .91 (language versions range: .89–.94).

#### Depression

Symptoms of depression were assessed with the Patient Health Questionnare-9 (PHQ-9 [38]). The PHQ-9 contains nine items rated on a scale of 0 (‘not at all’) to 3 (‘nearly every day’) which reflect the frequency with which participants have experienced the symptom in question within the previous 14 days. Based on the total sum (0–27), symptom severity can be divided into the categories ‘none-minimal’ (0–4), ‘mild’ (5–9), ‘moderate’ (10–14), ‘moderately severe’ (15–19), and ‘severe’ (20–27) depression. Participants with a sum score of > 14 were classified as having a depressive disorder. Cronbach’s α in the present study was α = .84 (.70 to .89 for the different language versions).

#### Somatization

Somatic symptoms were assessed with the Somatic Symptom Scale-8 (SSS-8 [39]). The SSS-8 is a shortened version of the PHQ-15 questionnaire developed for DSM-5 field trials. Each item can be rated on a 5-point Likert-Scale from ‘not at all’ to ‘very much’ referring to the previous 7 days. The total scores therefore range from 0 to 32, and are subdivided into five categories of severity: ‘none to minimal’ (0–3), ‘low’ (4–7), ‘medium’ (8–11), ‘high’ (12–15), and ‘very high’ (16–32) somatic symptom burden. A cut-off-score of > 11 was used for the present study. The internal consistency was α = .84 (.77 to .93 for the different language versions).

#### Self-rated mental and physical health

Participants were asked to rate their current mental and physical health on two visual analog scales ranging each from 0 to 100, on which higher scores indicate better health status.

### Statistical analyses

Statistical analyses were performed using the IBM SPSS statistical package, version 24.0 for Windows. Descriptive statistics were used to characterize the study sample. Six linear regression analyses using the Enter method were performed to look for potential predictors of somatization, anxiety, depression, PTSD, and self-rated mental and physical health symptoms among socio-demographic and flight-related variables. For all six models the following potential predictor variables were analyzed based on the conceptual framework presented above: (I) socio-demographic characteristics (age, sex, university degree, partnership, parenthood) and (II) flight-related characteristics (global peace index, flight duration, accompaniment during the flight, number of traumatic events, experience of at least one interpersonal traumatic event, current information about family members left behind, need for assistance with asylum application procedures, family reunion request and health care system).

## Results

### Sociodemographic, flight-related and mental health characteristics

Table 1 gives an overview of the study sample’s socio-demographic and flight-related characteristics. The mean age of the participants in the present study was 29.73 (SD = 8.79) years. The majority of the participants were male (*n* = 348, 69.3%). The largest groups were participants from Cameroon (*n* = 92, 18.3%), Venezuela (*n* = 85, 16.9%), and Syria (*n* = 52, 10.4%); all in all, participants from over 30 different countries took part in the survey. A bit more than half of the participants (*n* = 259, 51.9%) reported having a university degree. A total of 290 (57.9%) participants were single, 35.9% (*n* = 180) were married, 4.2% (*n* = 21) divorced, and 2% (*n* = 10) widowed, with 186 (37.1%) participants reporting that they have a partner and 201 (40.1%) that they have children. The mean flight duration was 1.9 years (SD = 3.2), with 44.7% (*n* = 224) of the participants reporting that they had been alone while fleeing. A total of 235 (46.9%) participants reported that they currently have no access to information about their family members. More than two-thirds (*n* = 349, 69.5%) of the participants expressed that they needed assistance in navigating the asylum application procedure, 141 (28.1%) said they needed help related to family reunion requests, and 196 (39%) reported needing help navigating the health care system.

**Table 1:** Sociodemographic and flight-related characteristics

	Participants ***N*** = 502
**Age**	
M/SD/Range	29.73/8.79/18–70
18–29 years	293 (58.3%)
30–39 years	142 (28.3%)
40–49 years	44 (8.8%)
> 50 years	23 (4.6%)
**Sex**	
male	348 (69.3%)
female	154 (30.6%)
**Country of origin**	
Cameroon	92 (18.3%)
Eritrea	41 (8.2%)
Iraq	23 (4.6%)
Nigeria	38 (7.6%)
Syria	52 (10.4%)
Turkey	43 (8.6%)
Venezuela	85 (16.9%)
other ^a^	128 (25.4%)
**University degree**^**1**^	
yes	259 (51.9%)
no	240 (48.1%)
**Last occupational status**^**2**^	
employed	107 (21.4%)
in retirement	4 (0.8%)
military service	23 (4.6%)
self-employed	121 (24.2%)
studies or training	108 (21.6%)
no employment	61 (12.2%)
other	77 (15.4%)
**Marital status**^**2**^	
single	290 (57.9%)
married	180 (35.9%)
divorced	21 (4.2%)
widowed	10 (2%)
**Partnership**^**2**^	
yes	186 (37.1%)
no	315 (62.9%)
**Parenthood**^**2**^	
yes	201 (40.1%)
no	300 (59.9%)
**Flight duration in years**	
M/SD/Range	1.9/3.2/0–27
**Accompaniment during the flight**^**2**^	
alone	224 (44.7%)
strangers	127 (25.3%)
friends	50 (10%)
family members	100 (20%)
**Information about family**^**2**^	
yes	266 (53.1%)
no	235 (46.9%)
**Support in Asylum Procedure**	
yes	349 (69.5%)
no	153 (30.5%)
**Support in Family Reunion**	
yes	141 (28.1%)
no	361 (71.9%)
**Support in Health Care System**	
yes	196 (39%)
no	306 (61%)

^1^*N* = 499; ^2^*N* = 501; ^a^ Country of origin other (N): Afghanistan (6), Algeria (2), Armenia (2), Belarus (1), Colombia (1) Ethiopia (20), Ghana (3), Georgia (7), India (2), Iran (5), Jordan (2), Kosovo (1), Kuwait (1), Lebanon (7), Liberia (1), Libya (13), Morocco (3), Myanmar (3), Palestine (10), Pakistan (7), Russian Federation (9), Senegal (2), Somalia (7), Sri Lanka (1), Tunisia (4), Ukraine (1), stateless (7)

Prevalence rates of somatization, depression, anxiety, and PTSD according to cut-off scores as well as mean scores for self-rated mental and physical health in total and stratified by sex are displayed in Table 2. Results on prevalence and comorbidity of somatization, depression and PTBS as well as traumatic events experienced by participants of this sample are described in more detail in Nesterko et al. [4].

**Table 2:** Symptom burden of anxiety, depression, somatization, and PTSD as well as self-rated mental and physical health in recently arrived refugees

	Female ***N*** = 154	Male ***N*** = 347	Total ***N*** = 501
**Somatization**			
SSS-8 cut off> 11	71 (46.1%)	86 (24.8%)	157 (31.3%)
**Depression**			
PHQ-9 cut-off> 14	37 (24.2%)^1^	71 (20.5%)	108 (21.6%)^4^
**Anxiety**			
HSCL-25 (subscale Anxiety) cut-off> 1.75	72 (47.7%)^2^	136 (39.2%)	208 (41.8%)^5^
**Symptoms of PTSD**			
PCL-5 cut-off> 32	58 (38.9%)^3^	114 (32.9%)	172 (34.7%)^6^
	**Female M / SD**	**Male M / SD**	**Total M / SD**
**Self-rated Mental Health**			
range 0–100	50.99 / 32.29	57.56 / 33.06	55.54 / 32.93
**Self-rated Physical Health**			
range 0–100	42.14 / 33.04	48.55 / 33.64	46.58 / 33.86

^1^*N* = 153; ^2^*N* = 151; ^3^*N* = 149; ^4^*N* = 500; ^5^N = 498; ^6^*N* = 496

### Predicting symptoms of somatization, depression, anxiety, and PTSD

Four separate linear regression analyses were performed to test which socio-demographic as well as flight-related factors are associated with symptoms of somatization, depression, anxiety, and PTSD (Table 3). Lack of current information about family members left behind and a subjective need for health care were found to be significant predictors for symptoms of depression (β = .17, *p* < .001; β = .15, *p* < .01), somatization (β = .13, *p* < .001; β = .24, *p* < .001), anxiety (β = .17, *p* < .001; β = .16, *p* < .01) and PTSD (β = .20, *p* < .001; β = .14, *p* < .01). Moreover, symptoms of somatization (β = −.19, *p* < .001), depression (β = −.10, *p* < .05), and anxiety (β = −.14, *p* < .01) were significantly associated with female sex. Being single was found to be a significant predictor for symptoms of depression (β = .10, *p* < .05). The number of different traumatic events experienced was significantly associated with symptoms of anxiety (β = .11, *p* < .05), and experiences of at least one interpersonal traumatic event were found to be a significant predictor for symptoms of PTSD (β = −.11, *p* < .05).

**Table 3:** Linear regression models predicting symptoms of somatization, depression, anxiety and PTSD

	Somatization (***N*** = 484)				Depression (***N*** = 483)				Anxiety (***N*** = 481)				Symptoms of PTSD (***N*** = 479)			
	B	SE	95% CI	β	B	SE	95% CI	β	B	SE	95% CI	β	B	SE	95% CI	β
Age	−.01	.04	−.09–.07	−.01	−.02	.04	−.10–.05	−.03	−.04	.04	−.12–.04	−.05	−.06	.11	−.29–.17	.03
Sex^1^	**−2.77**	**.65**	**−4.05 – −1.48**	−.19***	**− 1.39**	**.62**	**− 2.60 – -.17**	−.10*	**− 2.07**	**.65**	**−3.35 – -.79**	−.14**	− 2.88	1.89	−6.60 – .84	−.07
University degree^2^	.81	.64	−.44–.2.06	.06	.19	.60	−.99–1.36	.02	−.02	.63	−1.26 – 1.22	−.001	1.55	1.83	−2.06 – 5.15	.04
Partnership^3^	.67	.67	−.64–1.98	.05	**1.31**	**.63**	**.08–2.55**	.10*	1.13	.66	−.17–2.43	.08	2.01	1.92	−1.77 – 5.78	.05
Parenthood^4^	−1.25	.76	−2.75 – .25	−.09	−.83	.72	−2.25 – .58	−.06	−1.32	.76	−2.81 – .18	−.09	−1.37	2.21	−5.71 – 2.97	−.03
Global Peace Index	1.00	.76	−.49–2.50	.06	−.23	.71	− 1.63 – 1.18	−.01	−.02	.75	−1.50 – 1.46	−.001	−.88	2.18	−5.17 – 3.41	−.02
Flight duration	−.06	.10	−.25–.13	−.03	.10	.09	−.08–.27	.05	.05	.10	−.14–.24	.02	.48	.28	−.06–1.03	.08
Accompaniment during the flight^5^	−.43	.68	.1.76–.91	−.03	.22	.64	−1.03 – 1.48	.02	.59	.67	−.74–1.91	.04	−.05	1.95	−3.89 – 3.79	−.001
Number TE^a^	.06	.10	−.13–.26	.03	.16	.09	−.03–.35	.09	**.20**	**.10**	**−.001–.39**	.11*	.34	.29	−.24–.91	.06
Interpersonal TE^6^	−1.09	.89	−2.85 – .66	−.07	−.71	.84	−2.37 – .94	−.05	−.95	.89	−2.70 - .80	−.06	**−5.22**	**2.59**	**−10.31 – -.14**	−.11*
Information Family^6^	**1.86**	**.62**	**.64–3.08**	.13***	**2.15**	**.59**	**.99–3.30**	.17***	**2.30**	**.62**	**1.09–3.52**	.17***	**7.82**	**1.80**	**4.29–11.35**	.20***
Support in Asylum procedure^7^	.34	.70	−1.04 – 1.71	.02	.23	.66	−1.06 – 1.52	.02	.59	.69	−.77–1.95	.04	2.78	2.02	−1.18 – 6.74	.06
Support in Family Reunion^7^	−.99	.75	−2.46 – .49	−.06	.12	.71	−1.29 – 1.51	.008	.46	.75	−1.01 – 1.93	.03	.08	2.17	−4.19 – 4.34	.002
Support in Health Care System^7^	**3.38**	**.70**	**2.00–4.77**	.24***	**1.96**	**.66**	**.65–3.26**	.15**	**2.18**	**.70**	**.81–3.56**	.16**	**5.48**	**2.03**	**1.49–9.47**	.14**
**R**^**2**^	**.141**				**.110**				**.136**				**.135**			
**Adjusted R**^**2**^	**.115**				**.084**				**.110**				**.109**			
**F**	5.492***				4.153***				5.224***				5.188***			

*p < .05; **p < .01; ***p < .001; ^1^female = 1, male = 2; ^2^yes = 1; no = 2; ^3^partnership = 1, no partnership = 2; ^4^children = 1, no children = 2; ^5^unaccompanied = 1, accompanied = 2 ^6^yes = 1, no = 2; ^7^yes = 1, no = 0; ^a^Traumatic events

### Predicting self-rated mental and physical health

Finally, two separate linear regression analyses were conducted to test which factors are associated with self-rated mental and physical health (Table 4). Better self-rated physical health was significantly associated with male sex (β = .11, *p* < .05), university level education (β = −.11, *p* < .05), having a partner (β = −.12, *p* < .01), childlessness (β = .20, *p* < .001), lower number of different traumatic events experienced (β = −.12, *p* < .05), having current information about family members left behind (β = −.14, *p* < .01), and not having a subjective need for health care (β = −.10, *p* < .05). Better self-rated mental health was significantly associated with having a partner (β = −.15, *p* < .01), childlessness (β = .17, *p* < .01), lower number of different traumatic events experienced (β = −.15, *p* < .01), and having current information about family members left behind (β = −.18, *p* < .001).

**Table 4:** Linear regression models predicting self-rated mental and physical health

	Self-rated Physical Health (***N*** = 484)				Self-rated Mental Health (***N*** = 484)			
	B	SE	95% CI	β	B	SE	95% CI	β
Age	.23	.19	−.14–.61	.06	.21	.20	−.19–.60	.05
Sex^1^	**7.70**	**3.15**	**1.51–13.89**	.11*	6.38	3.28	.07–12.82	.09*
University degree^2^	**−7.06**	**3.07**	**−13.08 – −1.04**	−.11*	−1.78	3.19	−8.05 – 4.48	−.03
Partnership^3^	**−8.09**	**3.21**	**−14.40 – − 1.78**	−.12**	**−10.30**	**3.34**	**−16.86 – −3.74**	−.15**
Parenthood^4^	**13.40**	**3.68**	**6.18–20.63**	.20***	**11.49**	**3.82**	**3.97–19.01**	.17**
Global Peace Index	−.65	3.66	−7.84 – 6.55	−.01	−1.93	3.81	−9.42 – 5.55	−.02
Flight duration	−.33	.46	−1.23 – .58	−.03	.12	.48	−.83–1.06	.01
Accompaniment during the flight^5^	1.58	3.27	−4.84 – 8.01	.02	−.92	3.40	−7.61 – 5.77	−.01
Number TE	**−1.08**	**.49**	**−2.04 – −.12**	-.12*	**−1.35**	**.51**	**−2.35 – -.36**	−.15**
Interpersonal TE^5^	−3.09	4.31	−11.56 – 5.37	−.04	−4.17	4.48	−12.98 – 4.64	−.05
Information Family^5^	**−9.11**	**3.00**	**−15.00 – −3.23**	−.14**	**− 12.48**	**3.12**	**− 18.60 – −6.35**	−.18***
Support in Asylum procedure^6^	2.80	3.37	−3.82 – 9.41	.04	1.10	3.50	−5.78 – 7.99	.01
Support in Family Reunion^6^	4.93	3.62	−2.18 – 12.05	.07	2.85	3.77	−4.56 – 10.26	.04
Support in Health Care System^6^	**−6.98**	**3.39**	**−13.64 - -.31**	−.10*	2.26	3.53	−4.67 – 9.20	.03
**R**^**2**^	**.116**				**.099**			
**Adjusted R**^**2**^	**.089**				**.072**			
**F**	4.379***				3.663***			

*p < .05; **p < .01; ***p < .001; ^1^female = 1, male = 2; ^2^yes = 1; no = 2; ^3^partnership = 1, no partnership = 2; ^4^children = 1, no children = 2; ^5^unaccompanied = 1, accompanied = 2 ^6^yes = 1, no = 2; ^7^yes = 1, no = 0

## Discussion

Socio-demographic and flight-related characteristics were analyzed as possible predictors for symptoms of PTSD, anxiety, depression, and somatization as well as self-rated physical and mental health in recently arrived refugees in Germany. First, the findings on mental distress in the present study are in line with previous research revealing high prevalence rates of common mental disorders in different refugee populations [9, 10, 26]. The regression analyses identified several risk factors that predict higher PTSD, anxiety, depression, and somatization symptom burdens and poorer self-rated mental and physical health. One of the strongest predictors for the different disorders studied was ‘*lack of current information about family members left behind’,* being also positively associated with lower scores of self-rated physical and mental health. This result, which first does not seem surprising, can be understood in varying or rather interacting ways. First, the lack of knowledge about the situation of loved ones indicates an ongoing worry about their lives, especially in conflict and post-conflict settings, that may trigger own traumatic experiences, thus leading to symptom burden. Second, separation from family members and supportive networks, in many cases forced, reduces social support and puts people at risk of social isolation. These constitute risk factors for mental distress in refugees [3, 6]. Future research is needed to investigate the relationship of mental distress and information about family members in refugees as well as to reveal the severity of such relationships for different mental disorders separately.

Traumatic events as risk factor for developing different mental disorders being extensively investigated in previous research [8], the present study found a comparatively small impact of traumatic experiences. ‘*Number of traumatic events*’ was positively associated with symptom burden for anxiety only and negatively associated with self-rated mental and physical health. ‘*Experiences of at least one of interpersonal traumatic events’* were found to be a significant predictor for symptoms of PTSD in line with previous research [15–17]. These few interactions might be explained by the great heterogeneity of participants regarding their country of origin, and consequently flight route and flight duration (e.g. participants from Cameroon vs. participants from Venezuela might have had different kinds and numbers of traumatic experiences throughout their flight).

Another significant predictor for all disorders investigated and for self-rated physical health was *‘need for support in health care system’*. This result is important because the assessment of subjective needs of refugees is still rarely carried out, both in research and in practice. Our analyses, however, constitute a clear indication of a significant correlation between subjective needs for health care and symptoms of different mental disorders which were assessed according to diagnostic criteria. In addition, especially in light of no such correlation with self-rated mental health, there is still need for in-depth investigation of symptom representations among mentally ill refugees from different cultural backgrounds. Also, socio-demographic characteristics were found to be significant predictors for self-rated health status in participants of the present study. For example, ‘*having a partner’* and ‘*childlessness’* were positively associated with better self-rated mental and physical health, possibly indicating the presence of social support provided by the partner and the absence of responsibility and ongoing worries for children under difficult conditions. In light of this, it remains unclear why these aspects are directly related to self-rated health and do not interact with symptom burden of the different mental disorders studied. Thus, research focusing on the relationship between subjective and rather objective mental health status in refugees is needed.

All in all, the factors included in the models explained a relatively small proportion of total variance in the present study. On the one hand, some important risk factors were shown to identify refugees who are suffering from symptoms of different mental disorders upon arrival and, on the other hand, the results indicate that some other relevant factors not investigated in the present study may have even more of an impact on symptoms of PTSD, anxiety, depression, and somatization. So far, the research available on the mental health in different refugee populations is based on data collected in high-income Western countries, making asylum-seekers living in those countries for month and even years the key study population. Unfortunately, there appears to be no other study available that has used a methodology similar to that of this study, which makes it difficult to discuss our results with respect to previous research. Moreover, we do not know of any epidemiologically robust study so far, that has focused on self-rated mental and physical health in refugees.

Although the present study has some major strengths – (I) epidemiological approach, (II) assessment of recently arrived refugees, considering the time frame of symptom burden and excluding long-term post-migration stressors, (III) application of standardized instruments that have been translated and back-translated into 11 different languages, and (IV) assessment of self-rated physical and mental health status, something which hasn’t been investigated in a comparable population before – there are some factors that limit the generalizability and interpretation of our results and consequently shed light on some implications for future research. The analyses conducted in the present study are based on cross-sectional data. Consequently, despite methodological strengths, the generalizability of the findings is somewhat limited due to the fact that: (I) the data reflect a specific wave of refugees recently arrived in Germany at the time of data collection (e.g. refugees from Venezuela, Cameroon, and Syria) whereas the vast majority of refugees worldwide are located in camps within their countries of origin or bordering regions and (II) no information can be derived with respect to long-term impact of the risk factors analyzed. Thus, future research is needed which focuses on refugees in different host countries using a longitudinal approach. In addition, future research should focus on differences in mental health outcomes due to different cultural settings, participants’ ethnic and/or religious affiliations, as well as with respect to possible measurement invariance across different language versions of instruments [40–43]. Moreover, future research is needed using both self-reported scales and clinical interviews as well as functional assessments to better detect those in urgent need for treatment.

## Conclusions

The results of the present study provide initial methodologically robust insights for research and health care services, which should aid in better identifying newly arrived refugees in need of psychosocial care. The refugees with the highest symptom burdens are those who currently have no information about family left behind, female refugees, and those who report needing health care. Moreover, the findings of the present study indicate no link between symptom burden and need for assistance with submitting a family reunion request or navigating the asylum procedure. It is therefore all the more urgent, also in the sense of fulfilling humanitarian obligations by the host countries, on the one hand, to address the question of how to provide health care for highly vulnerable groups within refugee populations as quickly as possible regardless their residential status and, on the other hand, to provide accurate and useful data on these topics to inform current debates taking place in politics and the media.

## Data Availability

The datasets generated and analyzed during the current study are not publicly available due to ongoing analyses in respect to other research questions, but are available from the corresponding author on reasonable request.
